# Supplementary material for: The Resistome of ESKAPEE Pathogens in Untreated and Treated Wastewater: A Polish Case Study
Source: Biomolecules. 2022 Aug 21;12(8):1160. doi: 10.3390/biom12081160 (PMC9405806; doi:10.3390/biom12081160)
Supplement: Supplementary file 1 [file biomolecules-12-01160-s001.zip › biomolecules-1829966-supplementary.pdf]

The Resistome of ESKAPEE Pathogens in Untreated and Treated Wastewater: A Polish Case Study

Jakub Hubeny[1], Ewa Korzeniewska[1], Sławomir Ciesielski[2], Grażyna Płaza[3], Monika Harnisz[1]

[1]Department of Water Protection Engineering and Environmental Microbiology, Faculty of Geoengineering, University of Warmia and Mazury in Olsztyn, Olsztyn 10-720, Poland

[2]Department of Environmental Biotechnology, Faculty of Geoengineering, University of Warmia and Mazury in Olsztyn, Olsztyn 10-709, Poland

[3] Environmental Microbiology Unit, Institute for Ecology of Industrial Areas, Katowice 40-844, Poland

Supplementary Materials

Table S1. Total read base and total read count of raw data in the analyzed samples.

Season	Sampling site	Total read base (throughput of raw data) [Gb]	Total read count of raw data [M]
June 2018	UWW1	16	107.2
	PC1	11.4	75.46
	MR1	18.4	128
	UWW1	22.8	156.7
	URW1	17.6	118.3
	DRW1	29.4	201.9
November 2018	UWW2	16.6	103.2
	PC2	17.4	121.3
	AS2	15.8	112
	MR2	18.4	128.3
	TWW2	15	101
	DRW2	14.8	97
March 2019	UWW3	16.6	110
	PC3	28.6	191.8
	AS3	15.2	102.3
	UWW3	18.4	122.4

Table S2. Abundance of ESKAPEE group members (genus level) in wastewater and river water samples (ppm). Averaged results from three seasons.

Genus		Sampling sites						
		UWW	PC	AS	MR	TWW	URW	DRW
Acinetobacter	Mean	111.581	78.056	0.564	0.304	1.499	0.101	0.141
	SD	52.020	23.406	0.473	0.000	1.562	0.000	0.038
Enterobacter	Mean	0.495	0.880	0.006	0.000	0.393	0.017	0.007
	SD	0.514	0.586	0.008	0.000	0.544	0.000	0.007
Enterococcus	Mean	5.443	3.713	0.093	0.008	0.164	0.042	0.020
	SD	5.510	0.947	0.074	0.000	0.163	0.000	0.020
Klebsiella	Mean	2.070	3.682	0.013	0.008	0.179	0.025	0.007
	SD	1.743	2.851	0.013	0.000	0.216	0.000	0.007
Pseudomonas	Mean	3.534	4.796	0.647	0.140	0.692	0.237	0.144
	SD	1.252	0.953	0.526	0.000	0.509	0.000	0.144
Staphylococcus	Mean	0.046	0.072	0.003	0.000	0.003	0.008	0.000
	SD	0.036	0.026	0.004	0.000	0.004	0.000	0.000
Escherichia	Mean	1.034	2.221	0.009	0.023	0.177	0.008	0.035
	SD	0.416	1.493	0.001	0.000	0.143	0.000	0.035

Sampling sites: UWW – untreated wastewater, PC – wastewater exiting from the primary clarifier, AS – wastewater from the activated sludge tank, MR – wastewater from the multifunctional reactor, TWW – treated wastewater, URW – river water collected upstream from the wastewater discharge point, DRW – river water collected downstream from the wastewater discharge point

*SD - standard deviation

Table S3. Abundance of ESKAPEE group members (species level) in wastewater and river water samples (ppm). Averaged results from three seasons.

Species		Sampling sites						
		UWW	PC	AS	MR	TWW	URW	DRW
Acinetobacter baumannii	Mean	1.166	1.619	0.014	0.004	0.012	0.000	0.007
	SD	0.825	0.558	0.008	0.000	0.017	0.000	0.007
Enterococcus faecium	Mean	0.021	0.025	0.009	0.000	0.013	0.008	0.000
	SD	0.016	0.012	0.004	0.000	0.014	0.000	0.000
Escherichia coli	Mean	1.031	2.207	0.009	0.016	0.176	0.008	0.035
	SD	0.417	1.485	0.001	0.000	0.142	0.000	0.035
Klebsiella pneumoniae	Mean	0.897	0.275	0.000	0.004	0.161	0.008	0.000
	SD	1.143	0.330	0.004	0.000	0.216	0.000	0.000
Pseudomonas aeruginosa	Mean	0.120	0.084	0.000	0.000	0.014	0.008	0.007
	SD	0.156	0.090	0.000	0.000	0.011	0.000	0.007
Staphylococcus aureus	Mean	0.006	0.004	0.000	0.000	0.000	0.000	0.000
	SD	0.009	0.006	0.000	0.000	0.000	0.000	0.000

Sampling sites as in Table S2.

*SD - standard deviation

Table S4. Calculated reduction in the abundance of ESKAPEE group members (genus and species levels) after the wastewater treatment process. Averaged results from three seasons.

Taxonomic units		Sampling sites		% of reduction
		UWW	TWW	
Acinetobacter	Genuses	111.581	1.499	98.66
Enterobacter		0.495	0.393	20.65
Enterococcus		5.443	0.164	96.99
Klebsiella		2.070	0.179	91.36
Pseudomonas		3.534	0.692	80.41
Staphylococcus		0.046	0.003	94.04
Escherichia		1.034	0.177	82.90
Acinetobacter baumannii	Species	1.166	0.012	99.00
Enterococcus faecium		0.021	0.013	35.44
Escherichia coli		1.031	0.176	82.95
Klebsiella pneumoniae		0.897	0.161	82.11
Pseudomonas aeruginosa		0.120	0.014	88.35
Staphylococcus aureus		0.006	0.000	100.00
			Average	80.99

Sampling sites as in Table S2.

Table S5. Abundance of ESKAPEE group members (genus level) in wastewater and river water samples (ppm) collected in three seasons.

Genus	UWW			PC			AS			MR			TWW			URW			DRW		
	J	N	M	J	N	M	J	N	M	J	N	M	J	N	M	J	N	M	J	N	M
Acinetobacter	97.388	181.192	56.164	107.169	77.139	49.859	0.273	0.188	1.232	-	0.304	-	3.660	0.020	0.817	0.101	-	-	0.178	0.103	-
Enterobacter	1.203	0.281	0.000	0.066	1.422	1.152	0.000	0.018	0.000	-	0.000	-	1.161	0.000	0.016	0.017	-	-	0.015	0.000	-
Enterococcus	3.335	12.994	0.000	2.637	4.942	3.561	0.055	0.196	0.029	-	0.008	-	0.386	0.000	0.106	0.042	-	-	0.040	0.000	-
Klebsiella	1.945	4.264	0.000	3.366	7.321	0.360	0.031	0.009	0.000	-	0.008	-	0.054	0.000	0.482	0.025	-	-	0.015	0.000	-
Pseudomonas	1.875	3.828	4.900	3.512	5.791	5.083	1.367	0.125	0.450	-	0.140	-	0.868	0.000	1.209	0.237	-	-	0.287	0.000	-
Staphylococcus	0.089	0.048	0.000	0.080	0.099	0.036	0.008	0.000	0.000	-	0.000	-	0.000	0.000	0.008	0.008	-	-	0.000	0.000	-
Escherichia	0.858	1.609	0.636	1.405	4.316	0.944	0.008	0.009	0.010	-	0.023	-	0.351	0.000	0.180	0.008	-	-	0.069	0.000	-

Sampling sites as in Table S2. Months of sample collection: J – June, N – November, M – March.

Table S6. Abundance of ESKAPEE group members (species level) in wastewater and river water samples (ppm) collected in three seasons.

Species	UWW			PC			AS			MR			TWW			URW			DRW		
	J	N	M	J	N	M	J	N	M	J	N	M	J	N	M	J	N	M	J	N	M
Acinetobacter baumannii	1.782	1.715	0.000	1.246	2.407	1.204	0.000	0.009	0.020	-	0.008	-	0.035	0.000	0.000	0.000	-	-	0.015	0.000	-
Enterococcus faecium	0.023	0.039	0.000	0.013	0.041	0.021	0.000	0.009	0.010	-	0.000	-	0.032	0.000	0.008	0.008	-	-	0.000	0.000	-
Escherichia coli	0.849	1.609	0.636	1.391	4.291	0.938	0.008	0.009	0.010	-	0.023	-	0.348	0.000	0.180	0.008	-	-	0.069	0.000	-
Klebsiella pneumoniae	0.182	2.510	0.000	0.742	0.037	0.047	0.008	0.000	0.000	-	0.000	-	0.016	0.000	0.466	0.008	-	-	0.000	0.000	-
Pseudomonas aeruginosa	0.340	0.010	0.009	0.212	0.021	0.021	0.000	0.000	0.000	-	0.000	-	0.026	0.000	0.016	0.008	-	-	0.015	0.000	-
Staphylococcus aureus	0.000	0.019	0.000	0.013	0.000	0.000	0.000	0.000	0.000	-	0.000	-	0.000	0.000	0.000	0.000	-	-	0.000	0.000	-

Sampling sites as in Table S2. Months of sample collection: J – June, N – November, M – March.

Table S7. Selected subtypes and/or types of antibiotic resistance genes in a metagenomic analysis.

Genes	Types or subtypes
*bla*PER	1, 2
*bla*VEB	1
*bla*IMP	1
*bla*VIM	1, 2
*bla*GES	2, 4, 5, 6, 14, 18, 20
*bla*KPC	1, 3, 4, 5, 6, 7, 8, 9, 10, 11, 12, 13, 14, 15, 16, 17, 19, 22, 24
*bla*NDM	1, 2
*bla*OXA	23, 24, 51, 58
*bla*CTX-M	1, 10, 100, 101, 102, 103, 104, 105, 106, 107, 108, 109, 11, 110, 111, 112, 113, 114, 115, 116, 117, 12, 121, 122, 123, 124, 125, 126, 129, 13, 130, 131, 132, 134, 136, 137, 139, 14, 141, 142, 144, 147, 148, 15, 152, 155, 156, 157, 158, 159, 16, 160, 17, 19, 2, 20, 21, 22, 23, 24, 25, 26, 27, 28, 29, 3, 30, 31, 32, 33, 34, 35, 36, 37, 38, 39, 4, 40, 41, 42, 43, 44, 45, 46, 47, 48, 49, 5, 50, 51, 52, 53, 54, 55, 56, 58, 59, 6, 60, 61, 62, 63, 64, 65, 66, 67, 68, 69, 7, 71, 72, 74, 75, 76, 77, 78, 79, 8, 80, 81, 82, 83, 84, 85, 86, 87, 88, 89, 9, 90, 91, 92, 93, 94, 95, 96, 98, 99
*bla*SHV	2, 20, 21, 22, 23, 24, 25, 26, 27, 28, 29, 2A, 3, 30, 31, 32, 33, 34, 35, 36, 37, 38, 4, 40, 41, 42, 43, 44, 45, 46, 48, 49, 5, 50, 51, 52, 53, 55, 56, 57, 59, 6, 60, 61, 62, 63, 64, 65, 66, 67, 69, 7, 70, 71, 72, 73, 74, 75, 76, 77, 78, 79, 8, 80, 81, 82, 83, 84, 85, 86, 89, 9, 92, 93, 94, 95, 96, 97, 98, 99
*bla*TEM	3, 30, 33, 34, 4, 40, 42, 43, 45, 47, 48, 49, 52, 53, 54, 55, 57, 59, 6, 60, 63, 67, 68, 7, 70, 71, 72, 73, 75, 76, 78, 79, 8, 80, 81, 82, 83, 84, 85, 86, 87, 88, 89, 90, 91, 92, 93, 94, 95, 96
*qnr*B	1, 10, 11, 12, 13, 14, 15, 16, 17, 18, 19, 2, 20, 21, 22, 23, 24, 25, 26, 27, 28, 29, 3, 30, 31, 32, 33, 34, 35, 36, 37, 38, 4, 40, 41, 42, 43, 44, 45, 46, 47, 48, 49, 5, 50, 54, 55, 56, 57, 58, 59, 6, 60, 61, 62, 64, 65, 66, 67, 68, 69, 7, 70, 71, 72, 73, 74, 8, 9
qnrD	1, 2
qnrS	1, 10, 11, 13, 15, 2, 3, 4, 5, 6, 7, 8, 9
qnrC	
APH(2")-If	
APH(2")-Ig	
APH(2")-Ia	
AAC(3)-IV	

Sampling sites as in Table S2.

Table S8. Abundance of the analyzed antibiotic resistance genes associated with ESKAPEE bacteria in wastewater and river water samples (ppm). Averaged results from three seasons.

Gene		Sampling sites						
		UWW	PC	AS	MR	TWW	URW	DRW
bla$_{PER-1}$	Mean	0.098	0.071	0.000	0.000	0.014	0.000	0.000
	SD	0.022	0.005	0.000	0.000	0.008	0.000	0.000
bla$_{PER-2}$	Mean	0.036	0.016	0.000	0.000	0.000	0.009	0.000
	SD	0.017	0.008	0.000	0.000	0.000	0.000	0.000
bla$_{VEB-1}$	Mean	0.175	0.131	0.010	0.057	0.047	0.000	0.005
	SD	0.097	0.092	0.005	0.029	0.031	0.000	0.003
bla$_{IMP-1}$	Mean	0.009	0.000	0.010	0.029	0.032	0.000	0.013
	SD	0.004	0.000	0.005	0.021	0.029	0.000	0.003
bla$_{VIM}$*	Mean	0.092	0.025	0.000	0.000	0.000	0.000	0.000
	SD	0.018	0.010	0.000	0.000	0.000	0.000	0.000
bla$_{GES}$*	Mean	2.364	2.341	0.043	0.021	0.084	0.000	0.013
	SD	0.013	0.016	0.000	0.000	0.001	0.000	0.000
bla$_{KPC}$*	Mean	0.009	0.011	0.000	0.000	0.000	0.000	0.000
	SD	0.000	0.000	0.000	0.000	0.000	0.000	0.000
bla$_{NDM}$*	Mean	0.018	0.009	0.000	0.000	0.000	0.000	0.000
	SD	0.000	0.000	0.000	0.000	0.000	0.000	0.000
bla$_{OXA-23}$	Mean	0.019	0.013	0.000	0.000	0.000	0.000	0.000
	SD	0.014	0.006	0.000	0.000	0.000	0.000	0.000
bla$_{OXA-24}$	Mean	0.014	0.005	0.000	0.000	0.000	0.000	0.000
	SD	0.007	0.002	0.000	0.000	0.000	0.000	0.000
bla$_{OXA-51}$	Mean	0.025	0.019	0.000	0.000	0.000	0.000	0.000
	SD	0.011	0.009	0.000	0.000	0.000	0.000	0.000
bla$_{OXA-58}$	Mean	1.932	1.822	0.015	0.000	0.059	0.000	0.015
	SD	0.506	0.563	0.005	0.000	0.039	0.000	0.005
bla$_{CTX-M}$*	Mean	0.050	0.031	0.000	0.000	0.018	0.000	0.000
	SD	0.042	0.026	0.000	0.000	0.010	0.000	0.000
bla$_{SHV}$*	Mean	0.022	0.091	0.000	0.000	0.007	0.000	0.000
	SD	0.003	0.006	0.000	0.000	0.000	0.000	0.000
bla$_{TEM}$*	Mean	0.718	0.756	0.016	0.016	0.079	0.000	0.020
	SD	0.021	0.023	0.002	0.000	0.003	0.000	0.000
APH(2")-If	Mean	0.000	0.013	0.000	0.000	0.000	0.000	0.000
	SD	0.000	0.011	0.000	0.000	0.000	0.000	0.000
APH(2")-Ig	Mean	0.018	0.016	0.000	0.000	0.000	0.000	0.000
	SD	0.000	0.000	0.000	0.000	0.000	0.000	0.000
APH(2")-Ia	Mean	0.034	0.020	0.010	0.000	0.011	0.000	0.000
	SD	0.019	0.014	0.000	0.000	0.003	0.000	0.000
AAC(3)-IV	Mean	0.009	0.005	0.000	0.000	0.000	0.000	0.000
	SD	0.000	0.000	0.000	0.000	0.000	0.000	0.000
*qnr*B*	Mean	0.253	0.366	0.000	0.000	0.054	0.000	0.000

	SD	0.024	0.043	0.000	0.000	0.009	0.000	0.000
*qnr*C	Mean	0.009	0.016	0.000	0.000	0.000	0.000	0.000
	SD	0.000	0.000	0.000	0.000	0.000	0.000	0.000
*qnr*D*	Mean	0.042	0.037	0.000	0.000	0.000	0.000	0.000
	SD	0.000	0.000	0.000	0.000	0.000	0.000	0.000
*qnr*S*	Mean	1.084	1.455	0.019	0.000	0.107	0.009	0.009
	SD	0.264	0.345	0.000	0.000	0.024	0.000	0.003

Sampling sites as in Table S2.

* Genes were merged into type groups because subtype read counts were highly similar or did not differ significantly.

Table S9. Statistical analysis - Mann-Whitney U test.

Sampling site	Rank Sum	Rank Sum	U	Z	p-value
AS vs. WM5	897.5000	1055.500	401.5000	-1.10517	0.269088
MR vs. WM5	865.0000	1088.000	369.0000	-1.56272	0.118119

Table S10. Calculated reduction in the abundance of the analyzed antibiotic resistance genes after the wastewater treatment process. Averaged results from three seasons (ppm).

Gene	Sampling sites		% of reduction
	UWW	TWW	
bla$_{\text{PER-1}}$	0.098	0.014	85.70
bla$_{\text{PER-2}}$	0.036	0.000	100.00
bla$_{\text{VEB-1}}$	0.175	0.047	73.18
bla$_{\text{IMP-1}}$	0.009	0.032	-
bla$_{\text{VIM}}$	0.092	0.000	100.00
bla$_{\text{GES}}$	2.364	0.084	96.44
bla$_{\text{KPC}}$	0.009	0.000	100.00
bla$_{\text{NDM}}$	0.018	0.000	100.00
bla$_{\text{OXA-23}}$	0.019	0.000	100.00
bla$_{\text{OXA-24}}$	0.014	0.000	100.00
bla$_{\text{OXA-51}}$	0.025	0.000	100.00
bla$_{\text{OXA-58}}$	1.932	0.059	96.96
bla$_{\text{CTX-M}}$	0.050	0.018	63.73
bla$_{\text{SHV}}$	0.022	0.007	70.54
bla$_{\text{TEM}}$	0.718	0.079	89.02
APH(2")-If	0.000	0.000	-
APH(2")-Ig	0.018	0.000	100.00
APH(2")-Ia	0.034	0.011	68.49
AAC(3)-IV	0.009	0.000	100.00
*qnr*B	0.253	0.054	78.74
*qnr*C	0.009	0.000	100.00
*qnr*D	0.042	0.000	100.00

*qnr*S	1.084	0.107	90.13
		Average	91.09

Sampling sites as in Table S1.

Table S11. Abundance of the analyzed integron genes in wastewater and river water samples (ppm). Averaged results from three seasons.

Genes		Sampling sites						
		UWW	PC	AS	MR	TWW	URW	DRW
*int*I1	Mean	2911.291	2718.354	521.963	519.424	1061.185	78.177	178.985
	SD	373.284	1314.974	247.672	244.081	634.413	0.000	22.827
*int*I2	Mean	34.406	32.064	0.580	0.533	1.458	0.000	0.369
	SD	29.780	30.329	0.065	0.000	1.423	0.000	0.013
*int*I3	Mean	10.025	6.244	2.410	2.831	7.650	0.154	1.158
	SD	2.734	2.974	0.247	0.348	7.654	0.000	0.527

Sampling sites as in Table S2.

*SD - standard deviation

Table S12. Calculated pollution of the analyzed samples with antibiotic resistance genes after the wastewater treatment process. Averaged results from three seasons (ppm).

Gene	Sampling sites		Indication of pollution*
	URW	DRW	
bla$_{\text{PER-1}}$	0.000	0.000	-
bla$_{\text{PER-2}}$	0.009	0.000	↓
bla$_{\text{VEB-1}}$	0.000	0.005	↑
bla$_{\text{IMP-1}}$	0.000	0.013	↑
bla$_{\text{VIM}}$	0.000	0.000	-
bla$_{\text{GES}}$	0.000	0.013	↑
bla$_{\text{KPC}}$	0.000	0.000	-
bla$_{\text{NDM}}$	0.000	0.000	-
bla$_{\text{OXA-23}}$	0.000	0.000	-
bla$_{\text{OXA-24}}$	0.000	0.000	-
bla$_{\text{OXA-51}}$	0.000	0.000	-
bla$_{\text{OXA-58}}$	0.000	0.015	↑
bla$_{\text{CTX-M}}$	0.000	0.000	-
bla$_{\text{SHV}}$	0.000	0.000	-
bla$_{\text{TEM}}$	0.000	0.020	↑
APH(2")-If	0.000	0.000	-
APH(2")-Ig	0.000	0.000	-
APH(2")-Ia	0.000	0.000	-
AAC(3)-IV	0.000	0.000	-
*qnr*B	0.000	0.000	-
*qnr*C	0.000	0.000	-
*qnr*D	0.000	0.000	-
*qnr*S	0.000	0.004	↑

*int*I1	78.177	178.985	↑
*int*I2	0.000	0.369	↑
*int*I3	0.154	1.158	↑

Sampling sites as in Table S2.

* Indication of pollution: “-” no differences in gene abundance between sampling sites; “↓” a decrease in gene abundance after wastewater discharge; “↑” an increase in gene abundance after wastewater discharge.

Table S13. Abundance of the analyzed antibiotic resistance genes associated with ESKAPEE bacteria in wastewater and river water samples (ppm) collected in three seasons.

Gene	UWW			PC			AS			MR			TWW			URW			DRW		
	J	N	M	J	N	M	J	N	M	J	N	M	J	N	M	J	N	M	J	N	M
bla$_{PER-1}$	0.094	0.127	0.073	0.066	0.078	0.069	0.000	0.000	0.000	-	0.000	-	0.020	0.000	0.008	0.000	-	-	0.000	0.000	-
bla$_{PER-2}$	0.000	0.036	0.036	0.013	0.009	0.026	0.000	0.000	0.000	-	0.000	-	0.000	0.000	0.000	0.009	-	-	0.000	0.000	-
bla$_{VEB-1}$	0.160	0.064	0.300	0.132	0.017	0.243	0.000	0.010	0.000	-	0.057	-	0.020	0.000	0.074	0.000	-	-	0.005	0.000	-
bla$_{IMP-1}$	0.000	0.000	0.009	0.000	0.000	0.000	0.008	0.000	0.010	-	0.049	-	0.013	0.010	0.074	0.000	-	-	0.015	0.010	-
bla$_{VIM}$	0.061	0.132	0.082	0.020	0.035	0.021	0.000	0.000	0.000	-	0.000	-	0.000	0.000	0.000	0.000	-	-	0.000	0.000	-
bla$_{GES}$	2.358	2.356	2.379	1.983	3.153	1.889	0.033	0.076	0.010	-	0.008	-	0.145	0.049	0.059	0.000	-	-	0.005	0.020	-
bla$_{KPC}$	0.000	0.000	0.009	0.000	0.000	0.011	0.000	0.000	0.000	-	0.000	-	0.000	0.000	0.000	0.000	-	-	0.000	0.000	-
bla$_{NDM}$	0.000	0.000	0.018	0.000	0.009	0.000	0.000	0.000	0.000	-	0.000	-	0.000	0.000	0.000	0.000	-	-	0.000	0.000	-
bla$_{OXA-23}$	0.038	0.009	0.009	0.013	0.000	0.000	0.000	0.000	0.000	-	0.000	-	0.000	0.000	0.000	0.000	-	-	0.000	0.000	-
bla$_{OXA-24}$	0.009	0.018	0.000	0.000	0.000	0.005	0.000	0.000	0.000	-	0.000	-	0.000	0.000	0.000	0.000	-	-	0.000	0.000	-
bla$_{OXA-51}$	0.028	0.036	0.009	0.000	0.017	0.021	0.000	0.000	0.000	-	0.000	-	0.000	0.000	0.000	0.000	-	-	0.000	0.000	-
bla$_{OXA-58}$	1.595	2.647	1.555	1.073	2.430	1.964	0.000	0.019	0.010	-	0.000	-	0.093	0.000	0.025	0.000	-	-	0.010	0.020	-
bla$_{CTX-M}$	0.136	0.015	0.051	0.043	0.041	0.044	0.000	0.000	0.000	-	0.000	-	0.018	0.000	0.000	0.000	-	-	0.000	0.000	-
bla$_{SHV}$	0.030	0.000	0.015	0.092	0.160	0.011	0.000	0.000	0.000	-	0.000	-	0.007	0.000	0.000	0.000	-	-	0.000	0.000	-
bla$_{TEM}$	0.627	0.853	0.675	0.645	0.866	0.755	0.024	0.019	0.010	-	0.016	-	0.105	0.058	0.073	0.000	-	-	0.000	0.020	-
APH(2")-If	0.000	0.000	0.000	0.013	0.000	0.000	0.000	0.000	0.000	-	0.000	-	0.000	0.000	0.000	0.000	-	-	0.000	0.000	-
APH(2")-Ig	0.000	0.000	0.018	0.026	0.000	0.005	0.000	0.000	0.000	-	0.000	-	0.000	0.000	0.000	0.000	-	-	0.000	0.000	-
APH(2")-Ia	0.057	0.036	0.009	0.040	0.009	0.011	0.000	0.010	0.000	-	0.000	-	0.013	0.000	0.008	0.000	-	-	0.000	0.000	-
AAC(3)-IV	0.000	0.000	0.009	0.000	0.000	0.005	0.000	0.000	0.000	-	0.000	-	0.000	0.000	0.000	0.000	-	-	0.000	0.000	-
*qnr*B	0.564	0.129	0.066	0.731	0.237	0.129	0.000	0.000	0.000	-	0.000	-	0.045	0.000	0.062	0.000	-	-	0.000	0.000	-
*qnr*C	0.000	0.000	0.009	0.000	0.000	0.016	0.000	0.000	0.000	-	0.000	-	0.000	0.000	0.000	0.000	-	-	0.000	0.000	-
*qnr*D	0.038	0.000	0.045	0.026	0.000	0.048	0.000	0.000	0.000	-	0.000	-	0.000	0.000	0.000	0.000	-	-	0.000	0.000	-
*qnr*S	1.348	0.335	1.570	2.110	0.576	1.678	0.000	0.019	0.000	-	0.000	-	0.193	0.026	0.103	0.001	-	-	0.006	0.012	-

Sampling sites as in Table S2. Months of sample collection: J – June, N – November, M – March.

Table S14. Abundance of the analyzed integron genes in wastewater and river water samples (ppm) collected in three seasons.

Gene	UWW			PC			AS			MR			TWW			URW			DRW		
	J	N	M	J	N	M	J	N	M	J	N	M	J	N	M	J	N	M	J	N	M
*int*I1	3058.675	2486.815	3188.383	4231.258	2073.690	1850.114	692.015	346.832	697.093	-	346.832	-	1513.080	335.915	1334.561	78.177	-	-	162.844	195.127	-
*int*I2	26.576	67.319	9.324	19.895	66.588	9.709	0.000	0.533	0.626	-	0.533	-	2.464	0.000	0.451	0.000	-	-	0.360	0.378	-
*int*I3	10.683	12.370	7.022	8.795	6.960	2.978	3.077	2.585	2.235	-	2.585	-	16.484	3.463	3.004	0.154	-	-	0.786	1.530	-

Sampling sites as in Table S2. Months of sample collection: J – June, N – November, M – March.

Table S15. Spearman rank correlation analysis of experimental variables ($p < 0.01$).

	blaPER-1	blaPER-2	blaVEB-1	blaIMP-1	blaVIM	blaGES	blaKPC	blaNDM	blaOXA-23	blaOXA-24	blaOXA-51	blaOXA-58	blaCTX-M	blaSHV
blaPER-1	1.00	0.73	0.81	-0.23	0.88	0.91	0.84	0.88	0.88	0.88	0.88	0.91	1.00	0.96
blaPER-2	0.73	1.00	0.51	-0.78	0.88	0.51	0.84	0.88	0.88	0.88	0.88	0.57	0.73	0.68
blaVEB-1	0.81	0.51	1.00	-0.02	0.80	0.89	0.76	0.80	0.80	0.80	0.80	0.68	0.81	0.77
blaIMP-1	-0.23	-0.78	-0.02	1.00	-0.52	-0.05	-0.58	-0.52	-0.52	-0.52	-0.52	-0.14	-0.23	-0.29
blaVIM	0.88	0.88	0.80	-0.52	1.00	0.80	0.94	1.00	1.00	1.00	1.00	0.81	0.88	0.84
blaGES	0.91	0.51	0.89	-0.05	0.80	1.00	0.76	0.80	0.80	0.80	0.80	0.86	0.91	0.87
blaKPC	0.84	0.84	0.76	-0.58	0.94	0.76	1.00	0.94	0.94	0.94	0.94	0.76	0.84	0.88
blaNDM	0.88	0.88	0.80	-0.52	1.00	0.80	0.94	1.00	1.00	1.00	1.00	0.81	0.88	0.84
blaOXA-23	0.88	0.88	0.80	-0.52	1.00	0.80	0.94	1.00	1.00	1.00	1.00	0.81	0.88	0.84
blaOXA-24	0.88	0.88	0.80	-0.52	1.00	0.80	0.94	1.00	1.00	1.00	1.00	0.81	0.88	0.84
blaOXA-51	0.88	0.88	0.80	-0.52	1.00	0.80	0.94	1.00	1.00	1.00	1.00	0.81	0.88	0.84
blaOXA-58	0.91	0.57	0.68	-0.14	0.81	0.86	0.76	0.81	0.81	0.81	0.81	1.00	0.91	0.87
blaCTX-M	1.00	0.73	0.81	-0.23	0.88	0.91	0.84	0.88	0.88	0.88	0.88	0.91	1.00	0.96
blaSHV	0.96	0.68	0.77	-0.29	0.84	0.87	0.88	0.84	0.84	0.84	0.84	0.87	0.96	1.00
blaTEM	0.87	0.47	0.79	-0.04	0.76	0.82	0.80	0.76	0.76	0.76	0.76	0.90	0.87	0.91
APH(2")-If	0.45	0.45	0.41	-0.51	0.51	0.41	0.76	0.51	0.51	0.51	0.51	0.41	0.45	0.68
APH(2")-Ig	0.88	0.88	0.80	-0.52	1.00	0.80	0.94	1.00	1.00	1.00	1.00	0.81	0.88	0.84
aacA/aphD	0.94	0.63	0.78	-0.22	0.83	0.96	0.79	0.83	0.83	0.83	0.83	0.90	0.94	0.90
AAC(3)-IV	0.88	0.88	0.80	-0.52	1.00	0.80	0.94	1.00	1.00	1.00	1.00	0.81	0.88	0.84
QnrB	0.96	0.68	0.77	-0.29	0.84	0.87	0.88	0.84	0.84	0.84	0.84	0.87	0.96	1.00
QnrC	0.84	0.84	0.76	-0.58	0.94	0.76	1.00	0.94	0.94	0.94	0.94	0.76	0.84	0.88
QnrD	0.88	0.88	0.80	-0.52	1.00	0.80	0.94	1.00	1.00	1.00	1.00	0.81	0.88	0.84
QnrS	0.87	0.57	0.61	-0.31	0.76	0.86	0.80	0.76	0.76	0.76	0.76	0.92	0.87	0.91
intI1	0.91	0.51	0.89	-0.05	0.80	1.00	0.76	0.80	0.80	0.80	0.80	0.86	0.91	0.87
intI2	0.91	0.51	0.89	-0.05	0.80	1.00	0.76	0.80	0.80	0.80	0.80	0.86	0.91	0.87
intI3	0.87	0.37	0.89	0.22	0.67	0.93	0.58	0.67	0.67	0.67	0.67	0.77	0.87	0.79
Acinetoba	0.77	0.47	0.71	-0.25	0.76	0.89	0.80	0.76	0.76	0.76	0.76	0.85	0.77	0.81
Enterococ	0.87	0.68	0.63	-0.42	0.76	0.85	0.81	0.76	0.76	0.76	0.76	0.79	0.87	0.91
Escherichi	0.87	0.47	0.79	-0.04	0.76	0.82	0.80	0.76	0.76	0.76	0.76	0.90	0.87	0.91
Klebsiella	0.91	0.82	0.70	-0.35	0.81	0.72	0.76	0.81	0.81	0.81	0.81	0.69	0.91	0.87
Pseudomo	0.91	0.82	0.54	-0.40	0.81	0.67	0.76	0.81	0.81	0.81	0.81	0.83	0.91	0.87
Staphyloc	0.88	0.88	0.80	-0.52	1.00	0.80	0.94	1.00	1.00	1.00	1.00	0.81	0.88	0.84

	blaTEM	APH(2")-If	APH(2")-Ig	APH(2")-Ia	AAC(3)-IV	QnrB	QnrC	QnrD	QnrS	intI1	intI2	intI3	Acinetoba	Enterococ	Escherichi	Klebsiella	Pseudomo	Staphyloc
blaPER-1	0.87	0.45	0.88	0.94	0.88	0.96	0.84	0.88	0.87	0.91	0.91	0.87	0.77	0.87	0.87	0.91	0.91	0.88
blaPER-2	0.47	0.45	0.88	0.63	0.88	0.68	0.84	0.88	0.57	0.51	0.51	0.37	0.47	0.68	0.47	0.82	0.82	0.88
blaVEB-1	0.79	0.41	0.80	0.78	0.80	0.77	0.76	0.80	0.61	0.89	0.89	0.89	0.71	0.63	0.79	0.70	0.54	0.80
blaIMP-1	-0.04	-0.51	-0.52	-0.22	-0.52	-0.29	-0.58	-0.52	-0.31	-0.05	-0.05	0.22	-0.25	-0.42	-0.04	-0.35	-0.40	-0.52
blaVIM	0.76	0.51	1.00	0.83	1.00	0.84	0.94	1.00	0.76	0.80	0.80	0.67	0.76	0.76	0.76	0.81	0.81	1.00
blaGES	0.82	0.41	0.80	0.96	0.80	0.87	0.76	0.80	0.86	1.00	1.00	0.93	0.89	0.85	0.82	0.72	0.67	0.80
blaKPC	0.80	0.76	0.94	0.79	0.94	0.88	1.00	0.94	0.80	0.76	0.76	0.58	0.80	0.81	0.80	0.76	0.76	0.94
blaNDM	0.76	0.51	1.00	0.83	1.00	0.84	0.94	1.00	0.76	0.80	0.80	0.67	0.76	0.76	0.76	0.81	0.81	1.00
blaOXA-23	0.76	0.51	1.00	0.83	1.00	0.84	0.94	1.00	0.76	0.80	0.80	0.67	0.76	0.76	0.76	0.81	0.81	1.00
blaOXA-24	0.76	0.51	1.00	0.83	1.00	0.84	0.94	1.00	0.76	0.80	0.80	0.67	0.76	0.76	0.76	0.81	0.81	1.00
blaOXA-51	0.76	0.51	1.00	0.83	1.00	0.84	0.94	1.00	0.76	0.80	0.80	0.67	0.76	0.76	0.76	0.81	0.81	1.00
blaOXA-58	0.90	0.41	0.81	0.90	0.81	0.87	0.76	0.81	0.92	0.86	0.86	0.77	0.85	0.79	0.90	0.69	0.83	0.81
blaCTX-M	0.87	0.45	0.88	0.94	0.88	0.96	0.84	0.88	0.87	0.91	0.91	0.87	0.77	0.87	0.87	0.91	0.91	0.88
blaSHV	0.91	0.68	0.84	0.90	0.84	1.00	0.88	0.84	0.91	0.87	0.87	0.79	0.81	0.91	0.91	0.87	0.87	0.84
blaTEM	1.00	0.61	0.76	0.78	0.76	0.91	0.80	0.76	0.82	0.82	0.82	0.79	0.79	0.70	1.00	0.68	0.74	0.76
APH(2")-If	0.61	1.00	0.51	0.42	0.51	0.68	0.76	0.51	0.61	0.41	0.41	0.20	0.61	0.62	0.61	0.41	0.41	0.51
APH(2")-Ig	0.76	0.51	1.00	0.83	1.00	0.84	0.94	1.00	0.76	0.80	0.80	0.67	0.76	0.76	0.76	0.81	0.81	1.00
aacA/aphD	0.78	0.42	0.83	1.00	0.83	0.90	0.79	0.83	0.93	0.96	0.96	0.85	0.89	0.93	0.78	0.79	0.79	0.83
AAC(3)-IV	0.76	0.51	1.00	0.83	1.00	0.84	0.94	1.00	0.76	0.80	0.80	0.67	0.76	0.76	0.76	0.81	0.81	1.00
QnrB	0.91	0.68	0.84	0.90	0.84	1.00	0.88	0.84	0.91	0.87	0.87	0.79	0.81	0.91	0.91	0.87	0.87	0.84
QnrC	0.80	0.76	0.94	0.79	0.94	0.88	1.00	0.94	0.80	0.76	0.76	0.58	0.80	0.81	0.80	0.76	0.76	0.94
QnrD	0.76	0.51	1.00	0.83	1.00	0.84	0.94	1.00	0.76	0.80	0.80	0.67	0.76	0.76	0.76	0.81	0.81	1.00
QnrS	0.82	0.61	0.76	0.93	0.76	0.91	0.80	0.76	1.00	0.86	0.86	0.68	0.93	0.94	0.82	0.67	0.77	0.76
intI1	0.82	0.41	0.80	0.96	0.80	0.87	0.76	0.80	0.86	1.00	1.00	0.93	0.89	0.85	0.82	0.72	0.67	0.80
intI2	0.82	0.41	0.80	0.96	0.80	0.87	0.76	0.80	0.86	1.00	1.00	0.93	0.89	0.85	0.82	0.72	0.67	0.80
intI3	0.79	0.20	0.67	0.85	0.67	0.79	0.58	0.67	0.68	0.93	0.93	1.00	0.68	0.68	0.79	0.74	0.63	0.67
Acinetoba	0.79	0.61	0.76	0.89	0.76	0.81	0.80	0.76	0.93	0.89	0.89	0.68	1.00	0.85	0.79	0.50	0.56	0.76
Enterococ	0.70	0.62	0.76	0.93	0.76	0.91	0.81	0.76	0.94	0.85	0.85	0.68	0.85	1.00	0.70	0.79	0.79	0.76
Escherichi	1.00	0.61	0.76	0.78	0.76	0.91	0.80	0.76	0.82	0.82	0.82	0.79	0.79	0.70	1.00	0.68	0.74	0.76
Klebsiella	0.68	0.41	0.81	0.79	0.81	0.87	0.76	0.81	0.67	0.72	0.72	0.74	0.50	0.79	0.68	1.00	0.92	0.81
Pseudomo	0.74	0.41	0.81	0.79	0.81	0.87	0.76	0.81	0.77	0.67	0.67	0.63	0.56	0.79	0.74	0.92	1.00	0.81
Staphyloc	0.76	0.51	1.00	0.83	1.00	0.84	0.94	1.00	0.76	0.80	0.80	0.67	0.76	0.76	0.76	0.81	0.81	1.00

Sampling sites as in Table S2.

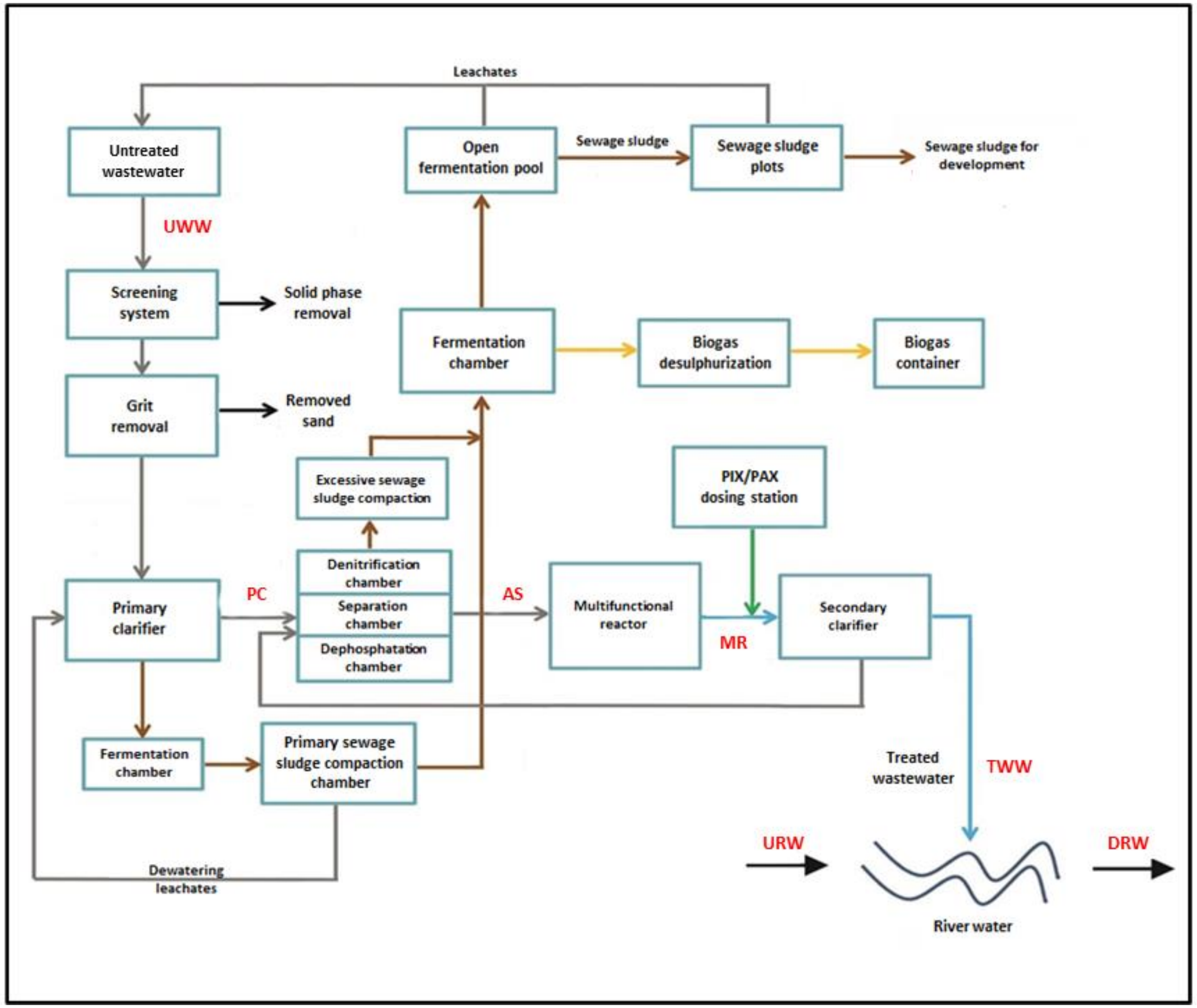

Figure S1. Diagram of the analyzed WWTP.

Sampling sites are marked in red.

Sampling sites as in Table S2.
